# An Ishmaelite

**Published:** 1888-04-07

**Authors:** Leslie Keith

**Affiliations:** Author of "The Chilcotes," "Uncle Bob's Niece," "East and West," etc.


### An Ishmaelite.

By Leslie Keith,

Author of "The Chilcotes," "Uncle Bob's Niece," "East and West," etc.

### Chapter I.—Father and Son.

For a great part of his life the friends of Mr. Joseph East gate had a way of saying that he ought to be a very happy man. There is often a little undernote of envy in a remark such as this, and when it was made by Mr. Eastgate's poor relations the envy was possibly more than hinted at; in some instances it was even accentuated. For Joseph had apparently everything that the heart of a reasonable man could desire: he was rich; he had a social position which, if not brilliant, was at least solid and respectable; and he had a pretty wife, who was universally pronounced to be amiable, and about whose irreproachable descent there could be no question. It was the opinion, however, of Mr. Eastgate's cousins down in the country—he was of a respectable Norfolk family—that Mrs. Eastgate was a little bit of a goose.

"That is what they mean when they talk so much of amiability," said cousin Fanny, the vicar's wife. "But, dear me, it's the privilege of people who have had Lady Blackrocks for their grandmothers, and who are wallowing in money, to be stupid. If I had only a quarter of Joseph's income!"

That was what they all said down in the country: the lawyer cousin, and the doctor cousin, and the maiden aunt, who lived on an annuity that would scarcely have kept Mrs. Joseph Eastgate in gloves and ribbons. "If I had a third of his wealth, what wouldn't I do for others! How generous I would be, how thoughtful, how tender to the poor and suffering!" Perhaps, and perhaps not. It seems a very easy thing to be virtuous when one's nobler qualities have a background of shining prosperity to set them off. But when it comes to the test, how is it? Does Dives bend to raise Lazarus to his side, and has that old simile of the needle's eye lost the meaning that it had when it was uttered eighteen hundred years ago?

It was rumoured that Joseph drew his purse-strings pretty tight when any appeal was made to him; and perhaps if Cousin Fanny had had the legacy she longed for left to her, she would still have scrimped the old women in ounces of tea, and bought stale buns for the school children's feast. Joseph, entirely indifferent to family criticism, was for a time almost as satisfied with his worldly condition as his friends professed to wish he should be. He had one of the handsomest houses in Portland Place, where, if the neighbourhood is a little less fashionable than other quarters, a great deal of solid comfort is still housed. It was there that his son was born; it was there too that his young orphaned nephew and niece came to live with him, and to share Master Frederick's nursery. Perhaps if the little ones had been the children of the lawyer, or the doctor, or the vicar down in the country, Mr. Eastgate would scarcely have been so generous towards them; but they were nearer in blood to him than the Norfolk kinsfolk, and, moreover, as the son and daughter of his deceased brother and late partner in the well-known firm of Eastgate and Moore, they were entitled to share a handsome fortune when they came of age. Uncle Joseph, in short, was the children's guardian, and the money, which was to accumulate during their minority, was safely invested where day by day it grew to more.

The little ones lived mostly on the top storey of the large house, and saw little of their uncle. He was a big, portly man, who had married in middle life and was much older than his wife. He had bushy black eyebrows that nearly met, and that gave him rather a fierce look, and a black beard, and a solid gold chain across his waistcoat. He had a heavy step; his boots creaked when he walked, and when on rare occasions the creaking boots were heard ascending the nursery stairs, little Helen Eastgate would run and hide under the table or behind the curtain. Very often Fred joined his cousin there, for they had in common a curious shrinking timidity which had its beginning, perhaps, in some fancied likeness between the pictures of Bluebeard in their nursery toy-books and the master of the house. It was only little Arthur who had the courage to go and hold his small face up to be kissed when the visitor came in.

"Where's Fred?" his father would say, in the loud voice that always sounded stern, scarcely noticing the other little man's overtures. And then the culprit was dragged forth by his nurse and prompted to say, "Good evening, papa."

Fred always said it as if he expected to be eaten the next moment, and the big brown eyes had the gaze of a frightened animal as they looked up into the dark face so far above him. As for Helen, nothing would induce her to become visible. She pressed her chubby hands against her ears, and held them there tight, quite sure that if she opened them she should hear Fred's groans as his head was being chopped off. This credulous, unreasoning terror was in some measure the outcome of nursery discipline; but Mr. Eastgate did not know this, and was sharply annoyed over his boy's sensitive distrust. Probably his own nerves had never quivered under the threat of a bogey. It was "Boney" who took the rôle in his days, as to an earlier generation across the Channel it was the English "Malbrook." In his own nursery he had the honour of playing the part of spectre himself. "You see what your papa"—or "uncle," as the case might be—"will say when he hears what a naughty child you've been," was generally sufficient to cause a prompt and trembling obedience from two, at least, of the little people there.

It was not a very happy nursery, though there were the handsomest toys in it that Cremer could supply. The authority at the head of it was as capricious as a weather-cock, and as variable of mood. When she was in a hurry to get down to the housekeeper's room, she either deprived her charges of the precious last hour of liberty by bundling them into their cribs—silencing all remonstrance by the argument of those bony knuckles that seemed made on purpose to render the process of face-washing a purgatory; or else she coaxed them to acquiescence and good behaviour by the promise of something nice to eat filched from the dinner going on down-stairs. On wet days, as she and her underling sat over the fire, they would entertain each other with ghastly tales of the *Police News* order, always with an oblique moral pointed at the children playing listlessly in a corner. Little Arthur was too young to suffer as yet, but the older boy and girl would draw nearer, fascinated by the grim recital, and would lie awake at nights peopling the awful darkness with visions of horror or crime, before which they shrank in terror: the boy whose downward career began with disobedience to his dear, kind nurse, and ended with the gallows; and the girl who fell down as dead as if you had shot her, after telling a lie. Was it any wonder that this cruel and vulgar method of exacting obedience turned the children—neither of them physically robust—into moral cowards?

The discipline of the nursery forbade the comfort of a night-light in that big room where the children slept. Little Arthur's crib was placed in the day nursery, where at least the fire-light in part kept the shadows at bay; but no such cheering gleam penetrated the inner room. It was some comfort to the little ones that they were still together.

"If nursey puts me alone in the little room, I'll die,'

sobbed Nellie, "like the little girl that died because she stole the sugar—I know I shall."

Fred could give her very little comfort; awful visions of his own fate hung over him. He could only put his hand out between the bars of his little bed and clasp Nellie's, that stole out to meet it. In that close grasp of each other, as if in defence of a common foe, nurse would sometimes find them when she came up from supper, their flushed faces on the pillow wearing an uneasy air even in sleep.

When Nellie grew a little older she was able to improve her position by the employment of certain small artifices. She was a timid, shrinking child, always weak and cowardly; but experience soon told her that a fib told in nurse's favour produced none of the direful consequences that the unvarnished truth entailed if it happened to be adverse to the nursery tyrant. She soon learned to coax, wheedle, and flatter; to be insincere because insincerity paid so much better than the openness which was indiscreet enough to carry tales. The one meant unlimited jam, the honour of sharing the society of nurse and under-nurse for an extra half-hour, and of listening to the tales of "'igh life" with which they were both seemingly conversant—perhaps, when nurse was very good-natured, the grace of a candle-end "to see to sleep by"; the other, disgrace, and banishment to the Erebus of the dark closet, where she suffered an agony from all her haunting fears.

The happiest times that the children knew were the hours spent with Mrs. Eastgate. She was a pretty little woman, small and fragile, with the same frightened, wistful look in her big dark eyes that was repeated in her own little boy, and in the younger Nellie, who was named for her aunt. She was a member of a family whose aristocratic record was no shield against poverty, and it was considered a very good match for her when she accepted the rich merchant; his family, if it could not equal hers, was at least respectable—nothing under a lawyer or a doctor for a generation or two, and his wealth was undoubted.

"You must take what you can get," said her grandmother, Lady Blackrock, who managed the family. "Your mother was sentimental and romantic, and see what has come of it! You must do better than that, my dear, and help your sisters too."

The old lady was very firm, and Helen's yielding was a foregone conclusion. There was no rebellion in that camp; if there had been someone else who had crept into the girl's thoughts, she wept a silent farewell to that ended dream in the darkness of the night, and came downstairs next morning to breakfast a little pale, but meek and obedient. She tried to be a good wife, and assuredly Joseph Eastgate considered himself to be a good husband. He was proud of his wife's beauty, and recognised the fitness of her submissive gentleness. He gave her very handsome presents of jewellery, and liked to see her prettily dressed, and he insisted that she should go much with him into the world—the world mostly of heavy solemn dinners, where she had not a word to say for herself, and felt inexpressibly bored.

It was generally when preparing for these functions that she had the little ones round her; sometimes she took them out driving with her, but Joseph did not like this.

"Can't you get another woman?" he would say. "There's your sister Arabella would jump at the chance. She doesn't often sit behind a pair of horses like mine."

But before her husband returned from the City, and after one of those dreary drives, either with Arabella or in solitary grandeur, she used to call the children to her dressing-room. Nellie would sit on the floor, and opening the jewel-cases, try on all the trinkets one by one; while the boys rummaged the little silver and china pots on the toilet-table for the chocolate she used to hide there for them.

"Why don't you come up to the nursery, auntie?" Nellie asked suddenly one day. "You never come there almost; are you afraid of nurse too?"

Mrs. Eastgate blushed as she answered hastily, "Isn't it nicer for you to be here, where we can be alone?"

But Nellie, who was a sharp little thing in spite of her timidity, was not deceived. "Auntie is afraid," she said to herself.

Nellie was soon to learn of another fear that had lodgment in the gentle lady's heart. One long-remembered day, when the children were assisting at the final touches Mrs. Eastgate's maid was putting to her mistress's toilet, Mr. Eastgate suddenly came into the room. A chilly silence fell on all the party at sight of his dark brow.

"Go downstairs," he said to the maid, "your mistress will ring when she wants you." The girl obeyed with alacrity, as if she were glad to escape from his presence.

"What is it, Joseph?" faltered Mrs. Eastgate, turning round a pale frightened face upon him.

"Nothing for you to interfere with, Helen," he said shortly. Then he turned to the children, who had stopped playing and were staring at him. "Who broke the statuette in the library?" he asked. His voice, always loud, sounded louder than ever—a voice of thunder. He looked Fred straight in the face with eyes that were very stern under the black shaggy brows.

The child met the look with a fascinated, terrified gaze. His face blanched till it was as white as Nellie's frock. He stood there a moment, spellbound, and then his lips slowly unclosed.

"Arthur," he said.

The little one thus accused looked up with a pair of wondering blue eyes, but his uncle took no notice of him.

"So my son can tell me a lie" he said in an awful voice. "*I saw you do it.* I saw you place the broken pieces together. If you had told me the truth I should have forgiven you. But now I mean to punish you. Go to your room and come down when I send for you."

Mrs. Eastgate started up with a smothered cry, but she was cowed by her husband's look, and sank down again, covering her face. The children got upstairs they scarce knew how, terror shaking their souls. The nurses were at tea downstairs; the emptiness of human presence in the big rooms accentuated the children's sense of desolation. There was no refuge to fly to. One has to put oneself back, as far as one can, into one's own childish days to realize what anguish such a moment may hold.

"I must go" said Fred, looking wildly in his little cousin's face, "or he will kill me; I suppose I'll die anyhow, like Ananias and Sapphira, because I've told a lie."

Nellie burst into a passion of weeping. "No, Fred," she said, "it's not true. I've told lies, heaps and heaps, and I'm living still."

When he had gone she crept down step by step to the lower landing, with feet that seemed weighted with lead. The door of her aunt's dressing-room was open, and Mrs. Eastgate came out and leaned upon the railings as if she could scarcely stand. The diamonds flashed in her hair, but her satin dress was not whiter than her face. At the sound of the little girl's lagging step she looked up and held out her arms. Nellie flew to them, and they clasped and clung to each other.

"Oh, Nellie, Nellie!" cried the poor mother, as Fred's cries came sounding up through the silent house, "my heart will break."

Nellie never forgot that hour. In after years she understood it all better. And then she said to herself, "Aunt Helen is afraid of him too."

*(To be continued.)*

